# Comparative Study of the Nutritional and Chemical Composition of New Oil Rape, Safflower and Mustard Seed Varieties Developed and Grown in Serbia

**DOI:** 10.3390/plants12112160

**Published:** 2023-05-30

**Authors:** Zorica S. Stojanović, Dajana D. Uletilović, Snežana Ž. Kravić, Žarko S. Kevrešan, Nada L. Grahovac, Ivana S. Lončarević, Ana D. Đurović, Ana M. Marjanović Jeromela

**Affiliations:** 1 Faculty of Technology Novi Sad, University of Novi Sad, Bulevar Cara Lazara 1, 21000 Novi Sad, Serbia; uletilovic.12.15.d@uns.ac.rs (D.D.U.); sne@uns.ac.rs (S.Ž.K.); ivana.radujko@tf.uns.ac.rs (I.S.L.); ana.djurovic@uns.ac.rs (A.D.Đ.); 2 Institute of Food Technology in Novi Sad, University of Novi Sad, Bulevar Cara Lazara 1, 21000 Novi Sad, Serbia; zarko.kevresan@fins.uns.ac.rs; 3 Institute of Field and Vegetable Crops, Maksima Gorkog 30, 21000 Novi Sad, Serbia; nada.grahovac@nsseme.com (N.L.G.); ana.jeromela@ifvcns.ns.ac.rs (A.M.M.J.)

**Keywords:** oilseeds, safflower, mustard, rapeseed, nutritional value, chemical composition

## Abstract

Oilseed crops are widely cultivated and are related to nutrition and human health as valuable nutraceutical sources with valuable biological properties. The growing demand for oil plants used in human and animal nutrition or for the processing industry has contributed to the diversification and development of a new variety of oil crops. Increased oil crop diversity, besides ensuring reduced sensitivity to pests and climate conditions, has also led to improved nutritional values. In order to enable oil crop cultivation to become commercially sustainable, a comprehensive characterization of newly created varieties of oilseeds, including their nutritional and chemical composition, is required. In this study, two varieties of safflower and white and black mustard were investigated as alternative oil species for nutritional parameters, mainly protein, fat, carbohydrate, moisture, ash, polyphenols, flavonoids, chlorophylls contents, acids and mineral composition, and compared with those of two different genotypes of rapeseeds as a traditional oil crop plant. The proximate analysis found that the highest oil content was found in the oil rape NS Svetlana genotype (33.23%), while the lowest was in black mustard (25.37%). The protein content varies from around 26% in safflower samples to 34.63%, determined in white mustard. High content of unsaturated fatty acids and low content of saturated fatty acid was observed in the analyzed samples. In mineral analysis, the dominant elements were phosphorus, potassium, calcium and magnesium, in descending order. The observed oil crops are also good sources of microelements, including iron, copper, manganese and zinc, accompanied by high antioxidant activity due to the presence of significant amounts of polyphenolic and flavonoid compounds.

## 1. Introduction

Over the past decade, a variety of oil crops have attracted more attention due to their potential use in human nutrition, primarily as good sources of nutrients. The wide application of oilseeds has led to the development of new genotypes and varieties of oil plants with the primary role of supplying the world population with sufficient oils. Nowadays, oil crops are being modified for high nutrition and improved oil quality, as well as to accommodate environmental conditions. Among oilseed species, there are major and alternative oil plants used for different purposes.

Rapeseed (*Brassica napus*) is one of the world’s major oilseeds, and its oil is the third most commonly produced vegetable oil worldwide [1]. Rapeseed is used for food, remedies, cosmetics, production of biodiesel or various industrial applications [2]. Due to its low levels of saturated—and high content of monounsaturated and polyunsaturated—fatty acids (especially oleic and α-linolenic fatty acids), rapeseed oil is considered to be one of the healthiest vegetable oils [3]. Rapeseed is a good source of protein for both animal and human consumption, with a balanced profile of essential amino acids, high crude fiber content and minerals [4]. It also contains beneficial components such as phenols, phytosterols and tocopherols [5,6]. 

Safflower (*Carthamus tinctorius* L.), also known as a false saffron, is vastly cultivated for its flower petals of specific colors and high levels of oil (20–47%) [7,8]. Safflower oil is rich in unsaturated fatty acids, such as linoleic and oleic acid, and has a low proportion of saturated fatty acids [9]. Additionally, safflower oil is rich in natural antioxidants, and α-, γ- and β-tocopherol are the most common [10]. Safflower oil is used extensively in the food industry and as biodiesel, and it is also suitable for human consumption due to its unique attributes.

Mustard is known as one of the oldest condiments [11]. It is an annual plant that includes several crops, such as white (*Sinapsis alba* L.) and black mustard (*Brassica nigra* L.). Mustard seeds have a high fat content, which can be as much as 47%. The oil composition of each mustard species is distinctive [12]. Thus, a high oleic acid content is typical for white mustard, while the abundance of linoleic acid is most common for black mustard [12]. Due to its spicy and hot flavor, mustard oil is mostly used for cooking in Asian countries. Furthermore, mustard seed contains a variety of phenolic compounds [13]. After oil extraction, most of the phenolic components remain in the cake, so it can also be used for the recovery of these compounds with potential applications as nutraceuticals and food ingredients [13]. 

The cultivation of rapeseed, safflower and mustard has the advantages of low production cost. This makes them a viable alternative crop with multiple possible uses. However, all the mentioned oil crops belong to different species and are represented by a large diversity of cultivars developed in different countries, which can significantly affect the component contents in the plant and seed. The composition of certain substances varies with climatic conditions, soil type, maturity of the plant and variety. Although some oil crop varieties (including some from Serbia) have been previously analyzed for basic composition, there is limited or no reported information on the content of primary ingredients (ash, moisture, protein, carbohydrate and crude fat), polyphenols, chlorophyll or mineral compositions in the seeds of safflower or white and black mustard, as well as two rapeseed genotypes created and grown in Serbia. In this regard, further investigations are required into the varieties which have not been examined previously. The availability of such data would help to estimate the quality of oil crops and to determine the influence of variety and species on the content of certain compounds in oilseeds. Therefore, this work is aimed at performing a proximate analysis to determine the polyphenols, chlorophylls and mineral contents in the seeds of two varieties of rapeseed and safflower and white and black mustard from Serbia. The results obtained from studying the nutritional and valorization potential of underused oil crops, particularly safflower and mustard seeds, provide valuable information on their potential uses in human nutrition and animal feeding. Additionally, this information may lead to the innovative use of these crops for various industrial purposes. With that overall goal, a comparison of nutritional composition among varieties and identification of varieties with improved nutritional characteristics accompanied by high phenolic content can help recognize oil crops with potentially greater health value for consumers.

## 2. Results and Discussion

### 2.1. Proximate Composition

To perform a systematic characterization of oil crop varieties created and registered in Serbia, a proximate analysis was first conducted. The proximate composition of the analyzed seed samples is given in Table 1. The major components of safflower seeds are carbohydrates, followed by fat and proteins. In white and black mustard samples, the dominant compounds are proteins and carbohydrates, followed by fats. As expected, the highest content of fat was found in rapeseed samples and was as high as 33.23%. These results are similar but lower than those presented by Gagour et al. [14] for rapeseed, who found that total oil content amounted to 38.8%. This difference may be primarily related to the ripeness degree of the seeds used in our study. Safflower had a higher fat content than white and black mustard and was close to the rapeseed fat content, suggesting that this alternative oil crops would be a suitable raw material for the oil industry. Previous examination of other safflower cultivars grown in Serbia reported oil content from 16.5 to 24.5%; varieties from Morocco contain up to 33.84% oil, while some cultivars from Turkey contain up to 38% oil [8,15,16].

Besides being good sources of fat, analyzed oil crop samples are rich in protein content, qualifying them as an excellent source of protein for humans and animals. 

### 2.2. Fatty Acid Composition

The fatty acid composition of an oil determines its nutritional and industrial properties and can affect its commercial value. The obtained results for fatty acid composition are shown in Table 2. No significant differences between varieties of investigated alternative oil crops (safflower and mustard) and two genotypes of rapeseeds as a traditional oil crop plant were observed (Table 2). Generally, all the analyzed oils are characterized by a high content of unsaturated fatty acids (86.42–95.08%) and a low content of saturated fatty acid (4.69–13.56%), which is desirable from a health perspective. Among unsaturated fatty acids, monounsaturated oleic acid (C18:1c) was the major component, with a content of 62.63 and 67.55% in the varieties NS Svetlana and Jovana, respectively, followed by essential polyunsaturated linoleic acid (18:2n6) and α-linolenic acid (18:3n3). The linoleic content was about 20%, while α-linolenic acid (18:3n3) was present in smaller quantities: 5–7% of total fatty acids. Results reported by other authors are similar to our data. Sagan et al. found that oleic acid was the dominant fatty acid in oil rape oil with a content of 55.22%, while the amounts of linoleic acid and linolenic acid were 24.24% and 10.34%, respectively [17]. From a nutritional point of view, n-6 and n-3 polyunsaturated fatty acids are important, as they have many beneficial health effects, and according to nutritional recommendation, their ratio should range from 1:1 to 4:1. In this aspect, examined rapeseed oils with an n-6/n-3 ratio of 2.95 and 3.52 could be considered as optimal. Furthermore, high oleic acid content indicates the suitable quality of these oil for cooking [18]. On the other hand, the analyzed varieties of rapeseed revealed a low content of erucic acid (below 1%): a fatty acid with negative health effects, which is discussed in more detail later. A wide range of erucic acid content has been published, depending on the variety [17,19], and its content is crucial for defining the usage of rapeseed oil. Safflower seed oil fatty acids are mainly composed of linoleic acid and oleic acid, which is consistent with previous studies [10,12]. These two unsaturated fatty acids represent approximately 85% of the total fatty acid content. The results show that a negligible amount of the other unsaturated fatty acids, namely α-linolenic acid, gondoic (20:1) and nervonic acid (C24:1), was detected in both varieties of safflower seed oils. Among saturated fatty acids, the most abundant were palmitic acid (6.37–7.27%), followed by stearic acid (3.01–4.75%), while the content of myristic, arachidic, behenic and lignoceric acid was under 1%. A previous study reported that oil of different safflower varieties contained 28.30–76.85% linoleic, 13.01–62.61% oleic and 5.96–7.05% palmitic acid as dominant fatty acids, while the content of stearic acid was in the range of 1.97–2.39% [20], indicating consonance with our results. The high content of essential linoleic acid (68–70%) makes safflower seed oils nutritionally valuable for human consumption. This polyunsaturated fatty acid is essential for normal growth and health promotion, as well as the prevention of coronary heart diseases, atherosclerosis and high blood pressure [21]. Although beneficial for human consumption, oils rich in PUFA are prone to oxidation, which leads to instability and short shelf life, and they are not suitable for cooking or frying [10]. 

White and black mustard oils are distinguished by their high erucic acid content, 46.13 and 41.82%, respectively, which is typical for the *Brassicaceae* family. Other authors have also confirmed the elevated level of erucic acid in mustard oil, with respective values of 43.46% and from 35.7 to 51.4% [22,23]. Erucic acid is a naturally occurring unsaturated fatty acid, and its formation is influenced by environmental conditions, irrigation measures and genetic engineering [24]. Negative health effects have been related to the consumption of food rich in erucic acid, such as myocardial lipidosis and heart lesions, which have been observed in laboratory rats [25]. Consequently, maximum levels of erucic acid in food have been established in Western countries [26,27], while mustard oil as a rich source of erucic acid is still often used for food preparation in Asian cuisine, especially in India [28]. To obtain a better ratio of fatty acid groups SFA:MUFA:PUFA (1:2:1) as well as bioactive substances, mustard oil with a high content of erucic acid is possible to blend with other conventional oils without erucic acid (sesame, sunflower, safflower, groundnut, soybean, olive oil, rice bran and palm) [29]. Rapeseed oil can also be a rich source of erucic acid, but the results from this study show that the investigated varieties showed low levels of this controversial fatty acid, which additionally makes them suitable for human consumption. White mustard seed oil can be used as a feedstock for biodiesel production and as an alternative fuel [30]. It can also be used in the production of biopolyols for the synthesis of rigid polyurethanepolyisocyanurate foams [31], edible biopolymer films for food packaging [32] and particle and interior boards, including furniture [33]. Additionally, nonedible white mustard seed oil can be used as a lubricant and for lighting purposes [30]. 

### 2.3. Minerals Composition

All analyzed samples contained significant amounts of important minerals essential for human nutrition (Table 3). Such mineral composition reveals the valuable potencies of all analyzed oil plants. The potassium and phosphorus contents were the highest, followed in descending order by calcium, magnesium and sodium. Trace elements of iron, zinc and manganese were present in similar content ranges in all analyzed samples, although statistically significant differences were observed in various oil plants. A significantly higher content of copper was detected in both varieties of safflower, while there were no statistically significant differences in copper contents between rapeseed NS Svetlana and black and white mustards. 

Comparable results have been reported previously by other authors [14,34]. Differences in mineral compositions may arise due to several factors, including the genetic properties of the plant species and environmental conditions in which it is grown. The genetic traits of plants may allow plants to take up and accumulate more certain minerals from the soil than others. Additionally, the mineral content of the soil in which the oil plant is grown can also affect its mineral composition [35]. If the soil is deficient in certain minerals, the oil plant may also have lower levels of those minerals. Furthermore, environmental factors such as temperature, precipitation, and soil pH value can affect the availability of minerals in the soil and thus impact the mineral composition of the seeds of oil plants. Hence, a combination of genetic and environmental factors can contribute to the variation in mineral composition observed among different plants and varieties of the same species.

### 2.4. Total Polyphenols Content, Total Flavonoids Content and Antioxidant Activity

The total polyphenol content (TPC) in analyzed samples ranged from 5.46 to 11.09 mg GAE/g DM (Table 4). A significantly higher TPC was obtained in white mustard, while the lowest TPC was found in black mustard. Nonsignificant differences in TPC were obtained between the two varieties of rapeseed and safflower, while differences in TPC among the species were statistically significant (*p* < 0.05). However, in the case of black mustard and both varieties of safflower, the difference in TPC was not significant. The obtained results are in agreement with previously reported data for safflower [8] and similar to reported data for rapeseed and mustard when adjusted for seed weight [13,36].

The total flavonoid content (TFC) was almost uniform and ranged from 4.56 QE/g DM to 6.11 mg QE/g DM (Table 4). Variation between TFC in different species was smaller than in the case of TPC. The only significant difference in TFC was obtained in the rapeseed variety Svetlana (6.11 mg QE/g DM) when compared with most plant species, even though there were no statistical differences between rapeseed varieties and white mustard. The present results regarding TFC are similar to those previously reported by other authors for all observed oilseeds [13,37,38]. 

Eventual differences could be caused by the difference in oilseed crops due to the different genetic potential of individual species for polyphenols biosynthesis. Apart from genetic reasons, different growing conditions and cultivation practices may also have a critical role in this respect. 

To evaluate the correlations between the antioxidant constituents (TPC and TFC) and the antioxidant activity assays (DPPH), as well as to identify the potential compounds which contribute to the antioxidant capacity of the observed oil crops, Pearson’s correlation coefficient was used. Table 5 shows the correlation among the variables, including DPPH assays and the antioxidant constituency of the oilseed spaces under investigation. 

Total polyphenolic compounds exhibit a close positive relationship with total flavonoids, thus explaining the Pearson coefficient of 0.6375 determined between TPC and TFC. The total polyphenolic compound content was moderately positively correlated with DPPH (r = 0.5236), while a significantly high correlation between total flavonoid content and DPPH assays was obtained (r = 0.9060). Since the antioxidant activity was attributed to the polyphenol compounds’ content, in the case of analyzed oil crops, it can be said that high antioxidant properties originate mainly from flavonoids present in samples. The obtained results are expected, since these compounds are well known to have high radical scavenging potential. Flavonoids are reported as the dominant polyphenolic compounds in most oilseeds as well [39]. 

### 2.5. Chlorophyll Contents

Chlorophyll content is connected to the maturity of the seeds. Typically, higher chlorophyll content is characteristic for seeds in the early stage, while during maturation it decreases significantly. Generally, in terms of oil stability, a high content of chlorophyll is not desirable, since it can act as a sensitizer for the photo-oxidation of oil. The color, flavor and oxidative stability of oil can be affected if oil is extracted from the seeds containing higher contents of chlorophyll pigments [40]. Consequently, additional costly processes should be applied for the removal of chlorophylls from such oil. Table 6 shows the results regarding chlorophyll a and b content in the analyzed seed samples. 

White and black mustard have the highest and statistically similar total chlorophyll contents (3.99 and 4.38 µg/g), followed by the NS Svetlana and Jovana rapeseed varieties, which contain 1.82 µg/g and 1.99 µg/g, respectively, while both varieties of safflower have the lowest chlorophyll content (1.72 and 1.33 µg/g). The content of chlorophyll in rapeseed was similar to previously reported values for the seeds in the last stage of maturity [41]. Since a maximum chlorophyll level of 12 mg/kg is allowable for top-grade rapeseed [42], the analyzed rapeseed varieties can be classified as the highest quality of oilseed. For other oilseeds, lower chlorophyll content is also desirable, which is also the case with the analyzed samples. 

## 3. Materials and Methods

### 3.1. Chemicals and Reagents

All chemicals used in this study were of analytical reagent grade. Quercetin, gallic acid and DPPH (1,1-diphenyl-2-picrylhydrazyl) were purchased from Sigma-Aldrich (St. Louis, MA, USA). Hydrochloric acid, sulfuric acid, hydroquinone and potassium dihydrogen phosphate were obtained from Merck (Darmstadt, Germany). Folin–Ciocalteu’s reagent was supplied from AppliChem (Darmstadt, Germany). Sodium sulfite, sodium carbonate, sodium hydroxide, aluminum chloride and ammonium heptamolybdate were purchased from Carl Roth (Karlsruhe, Germany). Methanol and n-hexane were from VWR Chemicals BDH (Fontenay-sous-Bois, France), while sodium nitrite was from Centrohem (Stara Pazova, Serbia). In all experiments, doubly distilled water was used.

### 3.2. Samples and Sample Preparation

**Figure 1:**
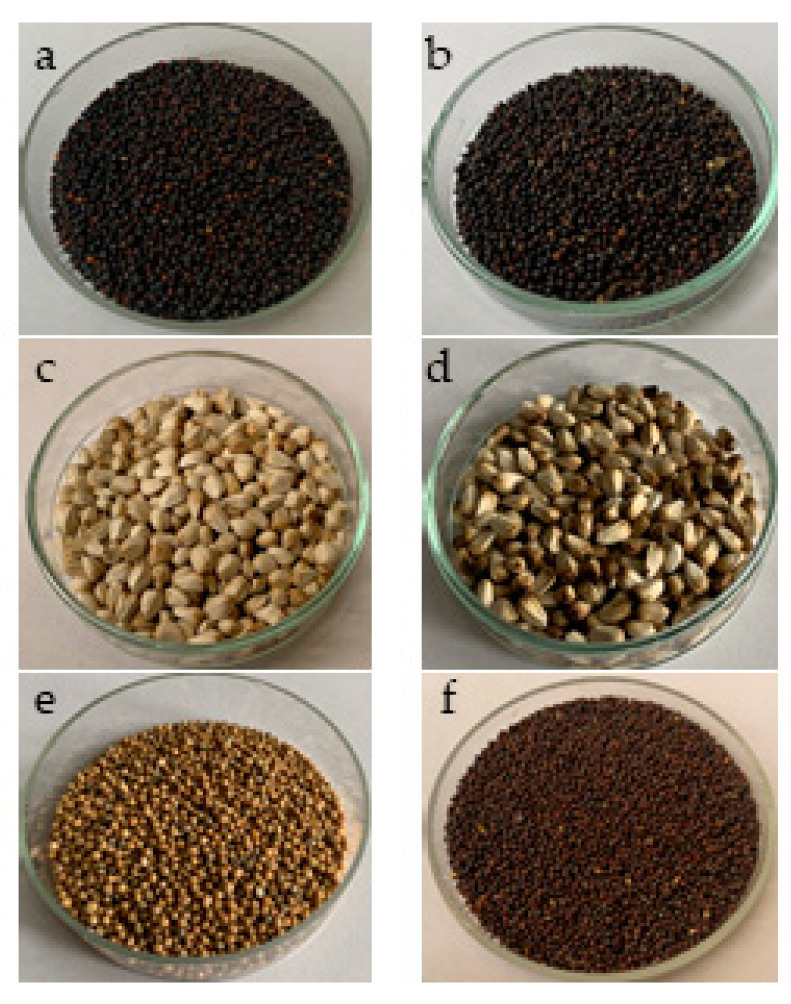
Analyzed seed samples: (**a**) rapeseed NS Svetlana, (**b**) rapeseed NS Jovana, (**c**) safflower NS Lana, (**d**) safflower NS Una, (**e**) white mustard NS Bela and (**f**) black mustard NS Crna.

The analyzed seed samples in this study included two different oil rape genotypes (*Brassica napus*), two genotypes of safflower (*Carthamus tinctorius* L.), white mustard (*Sinapis alba* L.) and black mustard (*Brassica nigra*) (Figure 1). NS Svetlana is a winter variety and Jovana is a spring variety of oil-seed rape. All analyzed varieties were created within the breeding program at the Institute of Field and Vegetable Crops in Novi Sad (Serbia) and registered by the Ministry of Agriculture, Forestry and Water Management of the Republic of Serbia (Table 7). Samples were gathered between 2020 and 2021. Before analysis, samples were grounded, passed through a 60-mesh sieve and stored in a refrigerator at −20 °C protected from light and moisture. 

### 3.3. Proximate Analysis

The proximate composition of oilseeds was determined using the recommended methods of AOAC International [43]. Dry matter values were determined by the oven-dry method. The moisture content was determined by weighing the samples in a quartz glass crucible (2 g) before and after drying at 105 °C until a constant mass (3 h). For ash determination, 2 g of the dried sample was placed in a porcelain crucible and incinerated in a muffle furnace at 550 °C until light gray ash was obtained. The crucibles were transferred to a desiccator and cooled to room temperature. The ash content was determined gravimetrically. For the determination of protein content, the Kjeldahl method was used. The protein percentage was calculated using a conversion factor of 6.25 [43]. Crude fat was determined by the Soxhlet method. A total of 5 g of the sample was extracted in a Soxhlet apparatus for 6 h using n-hexane as the extraction solvent at its boiling temperature. After extraction, the solvent was evaporated using a rotary evaporator R-200, Büchi (Zurich, Switzerland) at 40 °C until complete removal. The residue was dried at 70 °C until reaching constant weight (approximately 2 h), and afterward, crude fat content was determined gravimetrically. Total carbohydrate content was estimated by subtracting the difference between moisture, ash, crude fat and protein from one hundred percent.

The energy values were calculated by multiplying the mean values of fats, proteins and total carbohydrates using Atwater factors of 37 kJ/g (9.0 kcal/g), 17 kJ/g (4.0 kcal/g) and 17 kJ/g (4.0 kcal/g), respectively [44]. The results are expressed as kJ/100 g.

### 3.4. Fatty Acid Analysis

Lipids extracted from oilseeds were converted to fatty acid methyl esters (FAMEs) according to Kravić et al. [45,46] with minor modifications. In brief, 150 mg of lipid was dissolved in 2.4 mL of n-hexane, and an aliquot (0.6 mL) of 2 mol/L methanolic potassium hydroxide solution was added, vigorously shaken for 20 s and allowed to boil for 1 min in a water bath at 70 °C. After 20 s of shaking, 1.2 mL of 1 mol/L HCl was added, and the upper hexane phase containing the FAMEs was decanted and dissolved in hexane to 5 mL. The analysis of FAMEs was performed on an Agilent Technologies (Santa Clara, CA, USA) gas chromatograph model 7890B coupled with a 5977A mass selective detector with a DB-23 Agilent Technologies column (60 m × 0.25 mm i.d., film thickness 0.25 μm). Helium (purity 5.0) was used as carrier gas at a constant flow rate of 1.0 mL/min. The oven temperature program used for FAMEs separation was as follows: initial temperature 50 °C (held for 1 min) and a temperature increase of 25 °C/min to 200 °C followed by a second increase to 230 °C at the rate of 3 °C/min and kept under isothermal conditions for 7 min. The injector was maintained at 250 °C; the injection volume was 1.0 μL and the split ratio was 1:80. The mass spectrometer was operated in the electron ionization mode with the quadrupole temperature of 180 °C. Data acquisition was carried out in the scan mode (range 40–400 m/z); solvent delay time was 4.8 min. The content of each fatty acid expressed by mass percentage was calculated by the corrected peak area normalization method. A standard solution of a mixture of 37 FAMEs (37 component FAME Mix, 47885-U, Supelco) was used to define the correction factors.

### 3.5. Mineral Composition Analysis

Mineral content was determined using atomic absorption spectrophotometry (AAS). In brief, 2 g of the material was incinerated in a muffle furnace at 550 °C for 4 h, and the obtained ash was solubilized with 0.1 mol/L hydrochloric acid. The solution was filtrated using ash-free cellulose filter and analyzed using an atomic absorption spectrophotometer model ICE3000 (Thermo Fisher, Suzhou, China). All parameters for AAS (wavelength, slit and flame stoichiometry) were set following the manufacturer’s recommendation. The calibration curves used were in the linear range (R ≥ 0.998). 

Phosphorus was analyzed by spectrophotometry using the molybdenum blue method [47], with slight modifications in the sample preparation procedure. A dry sample (0.5 g) was digested using 5 mL of concentrated sulfuric acid and 15 drops of nitric acid. The mineralized sample was transferred to the volumetric flask, neutralized by adding several drops of 30% NaOH (1% solution of phenolphthalein was used for the indication), and finally diluted with doubly distilled water to 100 mL. An aliquot of 10 mL was transferred to the volumetric flask, 5 mL 5% ammonium heptamolybdate, 1 mL 20% sodium sulfite and 1 mL 0.5% hydroquinone were added, and the flask was filled with doubly distilled water. After 45 min, the absorbance was measured at 750 nm, and phosphorus content was calculated with a standard curve defined with KH_2_PO_4_.

### 3.6. Antioxidant Properties

An estimation of the total phenolic and flavonoid contents, as well as DPPH radical scavenging assay, were performed to evaluate the antioxidant properties of the studied samples. For all experiments, the extraction was initially carried out with 80% methanol using a sample-to-solvent ratio of 1:10 (*w/v*) in an ultrasound bath for 30 min. Afterward, the mixture was agitated on a shaker for 24 h at room temperature (25 ± 1 °C). During all procedures, extracts were protected from light by covering conical flasks with aluminum foil. The obtained extracts were centrifugated for 10 min at 6000 rpm, and the supernatant was filtered through a 45 µm filter (Macherey-Nagel, Düren, Germany). Crude methanolic extracts were used for further determination of total phenolics, total flavonoids and antioxidant activity. 

Total phenolic content was determined by the Folin–Ciocalteu method [22] with some modification. In a 10 mL volumetric flask, 150 µL of the extract was diluted with 6 mL of doubly distilled water, 500 µL of Folin–Ciocalteu’s reagent was added and the solution was mixed. After 60 s, 2 mL of 15% sodium carbonate was added, and the obtained solution was mixed for 30 s. In the end, the final volume of 10 mL was completed by adding doubly distilled water, and the mixture was allowed to stand for 45 min in a dark place. The absorbance was measured at 760 nm using a UV–Vis spectrophotometer (UV-2100 Unico). A calibration curve was defined using gallic acid as a standard, and results are expressed as mg gallic acid equivalents per gram of dry matter (mg GAE/g DM).

Total flavonoid content was determined using the aluminum chloride spectrophotometric method [48] with a slight modification. In a 10 mL volumetric flask, 150 µL of the extract was mixed with 4 mL of doubly distilled water and 0.3 mL of 5% sodium nitrite. After 5 min, 0.3 mL of 10% aluminum chloride solution was added and left for 1 min. Finally, 2 mL of 1 mol/L NaOH solution was added, and the final volume of 10 mL was completed with doubly distilled water. The obtained solution was mixed immediately. After incubation in the dark at room temperature for 15 min, absorbances were measured at 510 nm. For defining the calibration curve, standard solutions of quercetin were used, and total flavonoid content was expressed as mg quercetin equivalent per gram of dry matter (mg QE/g DM).

The potential antioxidant activity of the extracts was assessed by 2,2-diphenyl-1-picrylhydrazyl (DPPH) free radical assay [14] with a slight modification. A total of 25 µL of methanolic extract was diluted with 4 mL of doubly distilled water, and 800 µL 0.4 mmol/L methanolic DPPH solution was added. At the same time, a negative control was prepared by mixing 25 µL of 80% methanol with 800 µL 0.4 mmol/L methanolic solution of DPPH solution. The mixture was incubated in the dark for 30 min, and the absorbance was measured at 517 nm. Free radical inhibition of DPPH in percent was calculated using the following Equation (1):

(1) $$Inhibition\;of\;DPPH,\%=\frac{Ab-Aa}{Ab}\times 100$$

where Ab is the absorbance of blank (negative control), and Aa is the absorbance of sample/standard solution. A calibration curve was prepared using ascorbic acid standard solutions (50–500 mg/L). The results are expressed as milligrams of ascorbic acid equivalent per gram of dry matter (mg AAE/g DM). 

### 3.7. Estimation of Chlorophyll Content

The chlorophyll content was estimated according to the modified spectrophotometric method [49,50]. In total, 2 g of seed sample was vortexed with 20 mL of 96% methanol for 20 min. After extraction, the mixture was centrifugated at 6000 rpm for 10 min; the supernatant was collected and used for subsequent spectrophotometric analysis. Chlorophyll a and chlorophyll b were determined by measuring the absorbance at 666 nm and 653 nm and using the following equations [49,50]:

(2) $$Chlorophyll\;a(\frac{mg}{L})=15.65A_{666}-7.34A_{653}$$

(3) $$Chlorophyll\;b(\frac{mg}{L})=27.05A_{653}-11.21A_{666}$$

The content of Chlorophyll a and Chlorophyll b obtained were expressed as µg/g of dry matter.

### 3.8. Statistical Analysis

All analytical measurements were performed in triplicate, and the data were evaluated with Microsoft Office Excel (version 2007, Microsoft Corp, Redmond, WA, USA). Standard deviation (SD) was calculated in the case of all measurements. The calibration curves for all analytical techniques used were treated by linear regression, and the corresponding results are reported with a 95% confidence level. The data in tables are presented as the mean ± standard deviation calculated from values determined in three separate, repeated analytical runs. Statistical differences (*p*-value ≤ 0.05) among oil plant species and varieties were compared by employing a t-test. Pearson’s coefficient was used to evaluate the correlations between the antioxidant constituents and the antioxidant activity.

## 4. Conclusions

The conducted study has demonstrated that the examined oilseed crops represent prolific sources of numerous substantial nutrients, including oils, proteins, minerals and antioxidants. Regarding the oil content, there were no significant differences between varieties, while the difference was statistically significant among the seed species. Considering oil yields in alternative oilseeds, safflower with an oil content of around 28%, besides for human consumption, could be considered as a potential alternative to conventional rapeseed as industrial raw material for different purposes. The protein yield of oil rape and mustard seeds was remarkably affected by the variety, while for safflower varieties, this was not the case. The analysis of the oilseed samples revealed the highest levels of potassium, phosphorus and calcium. Iron, zinc and manganese were found to be the dominant microelements, whereas copper was the least abundant. Favorable fatty acid composition in rapeseed with a high content of oleic acid and low content of erucic acid enables its use in human nutrition. On the other hand, a high percentage of erucic acid in mustard oil limits its use in diet; however, a wide industrial application has been found. According to the polyphenol and flavonoid content, the observed oilseeds represent an excellent source of antioxidant compounds with a positive effect on human health. Between total flavonoid content and DPPH assays, a high correlation was obtained, indicating that antioxidant properties originated mainly from flavonoids present in the samples. It should be mentioned that the present study has some limitations, due to the fact that the nutritional and chemical composition may vary concerning the cultivating conditions, along with other agricultural practices of the crop, which may lead to further changes to the composition between varieties.

## Figures and Tables

**Table 1:** The content of primary ingredients in the studied oil crop seed samples.

Sample/Species	Proximate Analysis (g/100 g) ^1^					Energy ^2^ (KJ/100 g)
	Moisture	Ash	Proteins	Crude Fats	Carbohydrates	
Rapeseed NS Svetlana	5.44 ± 0.07 ^a,c,^*	4.44 ± 0.07 ^a,c^	21.58 ± 0.76 ^a^	33.23 ± 0.13 ^a^	35.31 ± 0.90 ^a^	2196.49
Rapeseed Jovana	5.98 ± 0.09 ^b,d^	4.35 ± 0.25 ^a,b,c^	27.21 ± 0.50 ^b^	32.28 ± 0.31 ^a^	30.18 ± 1.15 ^b^	2170.13
Safflower NS Lana	5.14 ± 0.08 ^c^	4.07 ± 0.13 ^a,b^	26.31 ± 0.64 ^b^	27.83 ± 0.32 ^b^	36.65 ± 1.01 ^a^	2100.08
Safflower NS Una	5.70 ± 0.07 ^a,d^	3.49 ± 0.20 ^b^	26.65 ± 0.58 ^b^	27.77 ± 0.26 ^b^	36.65 ± 0.60 ^a^	2099.06
White mustard NS Bela	6.27 ± 0.06 ^b^	5.00 ± 0.33 ^a,c^	34.63 ± 1.49 ^c^	25.41 ± 0.15 ^c^	28.69 ± 1.60 ^b^	2016.67
Black mustard NS Crna	5.87 ± 0.07 ^d^	5.09 ± 0.27 ^c^	28.12 ± 1.24 ^b^	25.37 ± 0.40 ^c^	35.55 ± 1.04 ^a^	2021.20

^1^ Average value ± SD, *n* = 3. ^2^ The energy content was based on At water factors. * Values with the same letter in a column are not significantly different at 5%.

**Table 2:** Fatty acid composition of the studied oil crops.

Fatty Acid	Fatty Acid Content (%) ^1^					
	Rapeseed		Safflower		White Mustard	Black Mustard
	NS Svetlana	Jovana	NS Lana	NS Una	NS Bela	NS Crna
14:0	-	-	0.08 ± 0.00	0.09 ± 0.00	^-^	^-^
16:0	4.29 ± 0.02	3.43 ± 0.19	6.37 ± 0.09	7.27 ± 0.02	2.24 ± 0.06	2.35 ± 0.07
16:1	0.15 ± 0.01	0.10 ± 0.01	^-^	^-^	0.09 ± 0.00	0.08 ± 0.00
18:0	1.35 ± 0.01	1.85 ± 0.01	3.01 ± 0.03	4.75 ± 0.02	0.72 ± 0.01	1.03 ± 0.05
18:1c	62.63 ± 0.52	67.55 ± 0.12	18.3 ± 0.14	17.56 ± 0.02	19.28 ± 0.42	13.64 ± 0.36
18:2n6c	20.97 ± 0.47	19.05 ± 0.34	70.06 ± 0.26	68.21 ± 0.06	8.93 ± 0.19	15.19 ± 0.16
18:3n3	7.10 ± 0.16	5.41 ± 0.10	0.26 ± 0.01	0.13 ± 0.02	6.45 ± 0.15	8.03 ± 0.04
20:0	0.64 ± 0.03	0.65 ± 0.02	0.54 ± 0.00	0.73 ± 0.00	0.57 ± 0.00	0.88 ± 0.01
20:1	1.54 ± 0.03	1.14 ± 0.05	0.33 ± 0.02	0.26 ± 0.01	9.79 ± 0.18	11.06 ± 0.05
20:2n6	-	-	-	-	0.23 ± 0.00	0.86 ± 0.01
20:3n3	-	-	-	-	-	0.12 ± 0.01
22:0	0.45 ± 0.05	0.34 ± 0.03	0.38 ± 0.02	0.44 ± 0.02	0.63 ± 0.02	0.90 ± 0.02
22:1	0.54 ± 0.11	0.17 ± 0.03	^-^	^-^	46.13 ± 0.50	41.82 ± 0.49
22:2	-	-	^-^	^-^	0.40 ± 0.01	0.85 ± 0.02
24:0	0.17 ± 0.12	0.31 ± 0.06	0.28 ± 0.07	0.28 ± 0.01	0.53 ± 0.10	0.66 ± 0.04
24:1	0.16 ± 0.11	^-^	0.41 ± 0.31	0.26 ± 0.02	4.01 ± 0.63	2.54 ± 0.29
SFA	6.9	6.58	10.66	13.56	4.69	5.82
UFA	93.09	93.42	89.36	86.42	95.08	93.33
MUFA	65.02	68.96	19.04	18.08	79.3	69.14
PUFA	28.07	24.46	70.32	68.34	15.78	24.19

^1^ Average value ± SD, *n* = 3. SFA—saturated fatty acids, UFA—unsaturated fatty acids, MUFA—monounsaturated fatty acids, PUFA—polyunsaturated fatty acids.

**Table 3:** Mineral compositions of the studied oil crops.

Mineral	Mineral Content (mg/100 g) ^1^					
	Rapeseed		Safflower		White Mustard	Black Mustard
	NS Svetlana	Jovana	NS Lana	NS Una	NS Bela	NS Crna
Na	25.53 ± 1.71 ^a,^*	26.22 ± 1.89 ^a^	37.36 ± 1.78 ^b^	15.32 ± 1.16 ^c^	26.72 ± 1.90 ^a^	32.86 ± 2.12 ^a,b^
K	1052.04 ± 30.66 ^a^	1094.96 ± 4.47 ^a^	1022.66 ± 67.65 ^a,b^	924.14 ± 31.83 ^b^	1195.04 ± 7.23 ^a^	1082.40 ± 19.13 ^a^
Ca	335.09 ± 0.40 ^a^	435.84 ± 1.46 ^b^	158.96 ± 8.03 ^c^	155.17 ± 3.48 ^c^	508.93 ± 30.45 ^b,d^	532.58 ± 12.26 ^d^
Mg	204.79 ± 2.12 ^a^	275.41 ± 1.75 ^b^	316.35 ± 10.89 ^c^	297.98 ± 17.95 ^b,c^	229.22 ± 3.24 ^d^	293.69 ± 6.79 ^b,c^
Zn	2.71 ± 0.10 ^a^	3.23 ± 0.09 ^b^	4.53 ± 0.30 ^c,d^	5.30 ± 0.09 ^d^	3.62 ± 0.05 ^c^	3.58 ± 0.47 ^b,c^
Fe	3.05 ± 0.22 ^a^	3.32 ± 0.03 ^a^	5.77 ± 0.37 ^b^	6.97 ± 0.01 ^c^	3.42 ± 0.13 ^a^	4.24 ± 0.17 ^d^
Mn	2.73 ± 0.33 ^a,c,d^	3.17 ± 0.18 ^a,d^	1.44 ± 0.09 ^b^	1.92 ± 0.12 ^c^	2.16 ± 0.11 ^c,d^	2.73 ± 0.18 ^d^
Cu	0.32 ± 0.03 ^a^	0.52 ± 0.04 ^b^	1.42 ± 0.05 ^c^	1.84 ± 0.01 ^d^	0.28 ± 0.01 ^a^	0.24 ± 0.01 ^a^
P	1282.58 ± 124.87 ^a^	843.82 ± 35.24 ^b^	1081.15 ± 71.77 ^c^	1172.23 ± 74.34 ^a,c^	907.07 ± 39.42 ^b^	1212.24 ± 108.39 ^a,c^

^1^ Average value ± SD, *n* = 3. * Values with the same letter in a row are not significantly different at 5%.

**Table 4:** Polyphenol and flavonoid compositions and antioxidant activity of the studied oil crops.

Seed Samples	Sample Genotype	Total Phenolic Compounds (mg GAE/g DM) ^1^	Total Flavonoid Compounds (mg QE/g DM) ^1^	DPPH (mg AAE/g DM) ^1^
Rapeseed	NS Svetlana	7.58 ± 0.28 ^a,^*	6.11 ± 0.21 ^a^	2.94 ± 0.03 ^a^
	Jovana	7.29 ± 0.11 ^a^	5.54 ± 0.46 ^a,b^	3.06 ± 0.10 ^a^
Safflower	NS Lana	5.97 ± 0.33 ^b^	5.08 ± 0.12 ^b^	1.85 ± 0.07 ^b^
	NS Una	5.46 ± 0.11 ^b^	5.06 ± 0.14 ^b^	2.06 ± 0.09 ^b,c^
White mustard	NS Bela	11.09 ± 0.28 ^c^	5.58 ± 0.23 ^a,b^	2.39 ± 0.14 ^c^
Black mustard	NS Crna	4.94 ± 0.31 ^b^	4.56 ± 0.22 ^b^	1.23 ± 0.04 ^d^

^1^ All values are presented as mean ± standard deviation (*n* = 3). * Means with different letters within a column are significantly different at *p* < 0.05.

**Table 5:** Pearson’s correlation coefficients between the variables, including the antioxidant constituents and antioxidant activity.

Variables	Pearson’s Coefficient
Total polyphenolics × Total flavonoids	0.6374 *
Total polyphenolics × DPPH	0.5236 *
Total flavonoids × DPPH	0.9060 **

* Correlation is significant at the 0.05 level. ** Correlation is significant at the 0.01 level.

**Table 6:** Chlorophyll a and b content in the studied oil crops.

Seed Samples	Sample Genotype	Chlorophyll a (µg/g DM) ^1^	Chlorophyll b (µg/g DM) ^1^	Chlorophyll Total (µg/g DM) ^1^
Rapeseed	NS Svetlana	0.94 ± 0.04 ^a,^*	0.88 ± 0.08 ^a^	1.82 ± 0.13
	Jovana	0.95 ± 0.04 ^a^	1.04 ± 0.14 ^a^	1.99 ± 0.10
Safflower	NS Lana	0.59 ± 0.04 ^b^	1.13 ± 0.08 ^a^	1.72 ± 0.13
	NS Una	0.35 ± 0.04 ^a^	0.98 ± 0.08 ^a^	1.33 ± 0.13
White mustard	NS Bela	1.69 ± 0.12 ^c^	2.30 ± 0.20 ^b^	3.99 ± 0.08
Black mustard	NS Crna	1.88 ± 0.13 ^c^	2.50 ± 0.02 ^b^	4.38 ± 0.15

^1^ Average value ± SD, *n* = 3. * Values with the same letter in a column are not significantly different at 5%.

**Table 7:** List of the analyzed seed samples with variety.

Seed Samples	Sample Genotype	Year of Registration ^1^
Rapeseed	NS Svetlana	2016
	Jovana	2007
Safflower	NS Lana	2019
	NS Una	2019
White mustard	NS Bela	2008
Black mustard	NS Crna	2008

^1^ Registered by the Ministry of Agriculture, Forestry and Water Management of the Republic of Serbia.

## Data Availability

Data will be made available on request.
